# Planning, prospective memory, and decision-making: three challenges for hierarchical predictive processing models

**DOI:** 10.3389/fpsyg.2012.00623

**Published:** 2013-01-18

**Authors:** Demis Basso

**Affiliations:** ^1^Faculty of Education, Free University of Bozen-BolzanoBolzano, Italy; ^2^Centro di Neuroscienze Cognitive ApplicateRome, Italy

**A commentary on**

**Whatever next? Predictive brains, situated agents, and the future of cognitive science**

by Clark, A. (2012). Behav. Brain Sci. (in press).

The present commentary on Clark (2012) will emphasize and discuss the role that high processing related to action planning may have in Bayesian predictive processing and will suggest possible directions for managing the issue.

Clark agrees to define planning as follows: “we imagine a future goal state as actual, then use Bayesian inference to find the set of intermediate states (which can now themselves be whole actions) that get us there” (§ 1.5). Although this definition was reported to corroborate the unified vision of sensor processing, motor control, and planning suggested by Toussaint (2009), it does not correspond to the current representation of the planning process in two aspects.

The first aspect entails the hierarchical vision of a plan as a succession of intermediate states. Research on planning (Basso et al., 2001; Phillips et al., 2001) has shown that the future goal state created in the beginning is accurate only in some particular circumstances (i.e., when both the task and algorithm are well-defined). In most cases, people are used to facing underspecified tasks in which a future goal state cannot be employed to derive the intermediate states (see Goel and Grafman, 2000). The first plan created is a general sketch of the intentions, a blurred image of the desired goal created by a feedforward prediction, which is successively refined through a continuous interaction between action and perception (Basso and Olivetti Belardinelli, 2006; Cisek, 2007). When the hierarchical predictive processing (HPP) presented by Clark is applied to the planning of motor sequences, it is undoubtedly convincing, as it explains the sensorimotor loop in an efficient way. However, the same mechanism may be too limited when applied to the planning of complex actions sequences. For example, in a planning task such as the Traveling Salesperson Problem (TSP: MacGregor and Ormerod, 1996), participants changed the initially planned strategy during task execution (Basso et al., 2006). Moreover, Cazzato et al. (2010) have demonstrated that a proficient performance is related to cognitive flexibility (shown while reconsidering the strategy chosen in the beginning) and not to the amount of information retrieved by ocular movements. That is, the overall organized behavior must be considered as more important than the summation of single actions.

The second aspect entails the timing in which the plan and (sequence of) action(s) may occur. In a stimulus-response paradigm a plan is created and promptly used because the action must be implemented as soon as possible. However, in many real-world situations, events develop in time and the planned actions must be postponed in order to be executed at the appropriate moment. Gärling (1994) has provided an example of everyday planning using a fictitious environment in which participants must organize a trip through several errands like stores with different opening hours. This delay between the plan and its implementation is also shared with other cognitive processes such as prospective memory (PM: Kliegel et al., 2008).

PM requires that a planned action should be executed only whenever the circumstances fulfill the conditions, which were commonly not present when the plan was created. In a standard scenario, a plan should be kept in mind while a person is involved in other ongoing activities, until the activating conditions are satisfied. At that moment, the person should inhibit the ongoing activity, switch to the prospective activity, and execute the plan. During the ongoing activity, some lure stimuli (*distracters*, see for PM: Bisiacchi et al., 2011) could share certain commonalities with the activating cues, but not enough to satisfy all the conditions. In these cases, according to HPP, the prospective action would increase its activation value and the probability of being executed as well, whereas it should be inhibited instead. HPP models are also required to account for the inhibition generated by long-lasting intentions. In the present state of the art, HPP-based models are outstanding in producing a response to a stimulus, but this response is locked in time to the stimulus itself (i.e., it is simultaneous to or must follow the stimulus as closely as possible). Providing explanations also for the realization of plans with delayed actions would be a good benchmark for determining the effective legitimacy of Clark's approach.

One attempt of this kind has been proposed by Shadlen et al. (2008) in decision-making. According to their accumulator model, decisions are taken when the accumulated evidence promoting a specific choice exceeds a certain threshold value (determined by prior information and costs). Abstract decisions (i.e., those producing a plan) are essentially aimed at creating rules instead of actions. It is important to highlight that, with respect to Clark's model, Shadlen's includes some components of the Bayesian inference, but it avoids using its major assumption, the posterior probability. Higher processing falls beyond the aim of Clark's target article, but it is central in its relevance for a model aiming at describing human processing. It is not that motor planning is more or less important than other higher-level planning, but they both show the same level of importance and need to be explained.

A unified mechanism for managing input–output processes is undoubtedly efficient and successful in species evolution. Therefore, it is likely that it is shared with many animals, too. However, since human beings have developed more complex behaviors and processes with respect to those managed by other animals, HPP must be shown to be flexible enough to (1) manage high levels of information (if it is the only mechanism), or (2) collaborate with other mechanisms of information processing. Clark is aware of this possible limitation, as he posed it in terms of open questions. The mechanism of predictive processing could be sufficiently powerful to be successfully applied to higher processing, as suggested by Cisek and Kalaska (2010, p. 276) too, but large-scale non-hierarchical mechanisms (able to recursively manage several processing systems) must also be hypothesized. Evidence from neuroscience enlightened that magnetic stimulation produces different effects, which are dependent on initial conditions of the cell assembly (Hoshi et al., 2000; Silvanto et al., 2008). State-dependent cell assemblies provide the cognitive system with flexibility that is likely to account for high-level (long-term) processes such as planning, PM, and decision-making.
